# MicroRNA-361-5p slows down gliomas development through regulating UBR5 to elevate ATMIN protein expression

**DOI:** 10.1038/s41419-021-04010-1

**Published:** 2021-07-28

**Authors:** Jiaoying Jia, Zhu Ouyang, Ming Wang, Wenjia Ma, Min Liu, Mingming Zhang, Mengqiang Yu

**Affiliations:** Department of Neurosurgery, The Second Xiangya Hospital of Central South University, Changsha, Hunan 410011 China

**Keywords:** Cell biology, Diseases

## Abstract

MicroRNA (miR)-361-5p has been studied to suppress gliomas development. Based on that, an insight into the regulatory mechanism of miR-361-5p in gliomas was supplemented from ubiquitin protein ligase E3 component N-recognin 5 (UBR5)-mediated ubiquitination of ataxia-telangiectasia mutated interactor (ATMIN). miR-361-5p, ATMIN, and UBR5 levels were clinically analyzed in gliomas tissues, which were further validated in gliomas cell lines. Loss/gain-of-function method was applied to determine the roles of miR-361-5p and UBR5 in gliomas, as to cell viability, migration, invasion, colony formation ability, and apoptosis in vitro and tumorigenesis in vivo. The relationship between miR-361-5p and UBR5 was verified and the interaction between UBR5 and ATMIN was explored. It was detected that reduced miR-361-5p and ATMIN and enhanced UBR5 levels showed in gliomas. Elevating miR-361-5p was repressive in gliomas progression. UBR5 was directly targeted by miR-361-5p. UBR5 can ubiquitinate ATMIN. miR-361-5p suppressed gliomas by regulating UBR5-mediated ubiquitination of ATMIN. Downregulating UBR5 impeded gliomas tumor growth in vivo. Upregulating miR-361-5p targets UBR5 to promote ATMIN protein expression, thus to recline the malignant phenotype of gliomas cells.

## Introduction

Gliomas form a heterogeneous group of central nervous system tumors [1] and are classified based on WHO Classification of CNS tumors published in 2016 [2]. Genetic risk factors, ionizing radiation, cellular phones, allergies and lifestyle, and diet are suggested as the risk factors for the occurrence and development of gliomas [3]. At present, variable therapies have been developing to treat the disease, and oncolytic virus therapy, stem cell therapy, immunotherapy, and electric field therapy are now on the market to improve the treatment prospects of recurrent high-grade gliomas [4]. The unsatisfactory outcome after treatment limits the survival of gliomas p authors are co-corresponding authoatients, thus an improved therapeutic approach is at wanting.

Effective delivery of miRNA alone or as part of a cytotoxic drug composition has been promising to improve clinical treatment of gliomas [5]. miR-361-5p is emerged as a regulatory actor in gliomas aerobic glycolysis, cell growth, and apoptosis [6]. Meanwhile, miR-361-5p stands for a repressor for matrix metalloproteinase (MMP)-2 transcription in gliomas migration and invasion [7]. Of significance, the process of epithelial-to-mesenchymal transition (EMT) in gliomas can be delayed by miR-361-5p targeting Twist1 [8]. Interestingly, a novel regulatory pattern of miR-361-5p in glioblastoma (GBM) implies its involvement in cancer progression and therapeutic potential [9].

Ubiquitin-proteasome system is a well-designed system that controls proteins by realizing a dynamic balance between ubiquitination and deubiquitination to control oncoproteins, tumor suppressors, and cell signaling pathways [10]. Ubiquitin protein ligase E3 component N-recognin 5 (UBR5) is a ubiquitin E3 ligase that mediates the degradation and ubiquitination of proteins involved in cancers [11, 12]. It has been testified that UBR5 interacts with ataxia-telangiectasia mutated (ATM) interactor (ATMIN) and enhances ubiquitination of ATMIN at lysine 238, thus weakening ATMIN interaction with ATM [13]. The oncogenic activities of ATMIN have been discussed in human cancer, such as head and neck cancer [14]. In GBM, inactivating ATMIN/ATM pathway ascribes to the inhibited cancer aggravation [15]. Though miR-361-5p, UBR5, and ATMIN have been mentioned in gliomas-related mechanisms, whether they could cooperate to modulate gliomas cell fate still lacks further exploration. Thus, this research was initiated to decipher miR-361-5p/UBR5/ATMIN axis in gliomas.

## Materials and methods

### Ethics statement

Experimental approval was gained from the ethics committee of The Second Xiangya Hospital of Central South University. A signed consent was provided by every participant. Animals were treated as the care and use of laboratory animals, and were approved by the animal research ethics committee of The Second Xiangya Hospital of Central South University.

### Clinical samples

Gliomas tissue samples were collected from 98 patients with gliomas who received general surgery in The Second Xiangya Hospital of Central South University, including 52 males and 46 females, 24–69 years old, with an average age of (46.5 ± 7.52) years old. Among them, 41 cases were in WHO grade 2, 32 cases in WHO grade 3, and 25 cases in WHO grade 4. Patients were confirmed with gliomas by pathological examination and the clinical data were complete. Any gliomas treatment was not performed in the past 3 months. Healthy brain tissues (*n* = 20) from the donors died of accidents were used as controls [16].

### Cell culture

GBM cell line U251 (XY-XB-1030, Shanghai XYBIO Biotechnology, Shanghai, China), the other GBM cell lines U87, LN229, and A172 (ATCC, VA, USA) and normal human astrocytes NHA (DC790, Shanghai Ze Ye Biotechnology Co., Ltd., Shanghai, China) were cultured in Dulbecco’s Modified Eagle Medium (DMEM, Invitrogen, CA, USA) supplemented with 10% fetal bovine serum (FBS, Hyclone, UT, USA) [17].

### Cell transfection

miR-361-5p mimic and inhibitor sequence and its corresponding negative control (NC) sequence dsRNA oligonucleotides were available from Ribobio (Guangzhou, China). U87 and U251 cells growing to 50% confluence were transfected with corresponding dsRNA oligonucleotides (2 μg) through 10 μL X-tremeGENE siRNA Transfection Reagent (Hoffmann-La Roche Ltd., Basel, Switzerland). The final concentration in transfection was 50 nM [7]. The siRNA Smartpool and RISCfree control siRNA against human and mouse UBR5 (Thermo Fisher Scientific, Massachusetts, USA) were transfected into U87 and U251 cells through Dharmafect 1 (Invitrogen) [13].

### Reverse transcription-quantitative polymerase chain reaction (RT-qPCR)

Total RNA was extracted from cells and frozen gliomas tissues with Trizol reagent (Invitrogen, Thermo Fisher). Gene-specific primers were used to synthesize miR-361-5p cDNA from total RNA. RT-qPCR determined gene expression via SYBR-Green PCR Master Mix kit. Gene mRNA levels were calculated by 2^−ΔΔCT^ method [18], and normalized to U6 and glyceraldehyde-3-phosphate dehydrogenase (GAPDH) [19]. Table 1 showed the primers.

**Table 1:** Primer sequences.

Primer sequences	Forward (5′→3′)	Reverse (5′→3′)
miR-361-5p	AGGGGTACGTCGTATCCAGT	GTATCCAGTGCGTGTCGTGG
UBR5	ACGAGAAGGAAAGCACCATG	CTTCTCAGAAACTTCTCGTAAC
ATMIN	AACAGCACTGCAGTCTCACA	CTGGTCTAGGGATTGGTTGGT
Bax	CCAGCTCTGAGCAGATCATG	TGCTGGCAAAGTAGAAAAGG
Bcl-2	GACTTCGCCGAGATGTCCAG	CAGGTGCCGGTTCAGGTACT
U6	GCACATTCTCCCCAGTTATGA	TCACAAATTTGCATGTCATCCT
GAPDH		GGTAACCAGGCGTCCGATA

*miR-361-5p* microRNA-361-5p, *UBR5* Ubiquitin protein ligase E3 component N-recognin 5, *ATMIN* ataxia telangiectasia mutated interactor, *Bax* Bcl-2-associated X, *Bcl-2* B cell lymphoma 2; GAPDH, glyceraldehyde-3-phosphate dehydrogenaseglyceraldehyde-3-phosphate dehydrogenase.

### Western blot assay

Total protein lysate was prepared by treating cells with radio-immunoprecipitation assay (RIPA) buffer (Beyotime, Shanghai, China) and centrifugation at 17,000 × *g*. The protein concentration was determined with a bicinchoninic acid kit (Beyotime, Nantong, China). The total protein lysate was denatured by 10% sodium dodecyl sulfate-polyacrylamide gel electrophoresis (SDS-PAGE), transferred to polyvinylidene fluoride membranes, and sealed with 5% skimmed milk. Anti-human UBR5 antibody (1:1000) was obtained from immunizing rabbits with human UBR5 peptide (KWSEPYRNAQNPS) [20]. The primary antibodies against UBR5, MMP-2 (ab37150, 1:1000; Abcam), ATMIN (ab3271, 1:5000; Millipore Sigma), Bax (ab32503, 1:1000, Abcam), Bcl-2 (ab182858,1:2000; Abcam) and GAPDH (ab9482, 1:1000; Abcam) were probed with the membrane. Subsequently, the membrane was further probed with horseradish peroxidase-conjugated secondary antibody (1:2000; Cell Signaling Technology, Boston, USA), developed using an enhanced chemiluminescence kit (Beyotime), and visualized by ChemImager5500 V2.03 software (Alpha Innotech, San Lean, USA). Using GAPDH as an internal control, the integrated density value of the protein band was quantified [20].

### Transwell assay

Cell invasion assay was performed with a 24-well Transwell (Corning, USA). The Transwell chamber was coated with BD Matrigel matrix (Corning), and U87 and U251 cells (3 × 10^4^) were seeded on the BD Matrigel in the upper chamber with serum-free medium. The lower chamber was filled with complete medium containing 10% FBS. Cell invasion was observed in six fields under a fluorescence microscope.

In the Transwell migration test, U87 and U251 cells (3 × 10^4^) were suspended in serum-free medium and placed in the upper chamber without BD Matrigel matrix. The lower chamber was supplemented with serum-free medium. After 36 h, cells were fixed with 4% paraformaldehyde and stained with 0.5% crystal violet [21].

### Colony formation assay

Cell suspension was cultured in 10% FBS-Roswell Park Memorial Institute-1640 for 10 d in a 6-well plate at 400 cells/well. Then, the colonies were fixed with anhydrous methanol and stained with 0.1% crystal violet. The colonies larger than 2 mm were counted [22].

### Flow cytometry

Annexin 8-fluorescein isothiocyanate (FITC)/propidium iodide (PI) method was adopted to detect cell apoptosis. Cells were incubated 6-well plates for 48 h, washed with 1× binding buffer (185 mL), and incubated with 5 µL AnnexinV FITC and 5 µL PI. Cell apoptosis was detected by FACSCalibur flow cytometer and Cell Quest Pro software (Thermo Fisher). The data were analyzed by FlowJo 7.6 software [23].

### 3-(4,5-dimethylthiazol-2-yl)-2,5-diphenyltetrazolium bromide (MTT) assay

After transfection of 24 h, cells were trypsinized and grew in 96-well culture plates at 3000 cells/well. Cultured for 0, 24, 48, and 72 h, cells were combined with MTT solution (10 µL, 5 mg/mL; Sigma-Aldrich, St. Louis, Missouri, USA) for another 4-h incubation, and reacted with dimethyl sulfoxide (150 µL). The absorbance was measured at 490 nm with a SpectraMax M3 microplate reader (Molecular Equipment Corporation, LLC, Sunnyvale, CA, USA). Each group set five replicates [24].

### Immunocoprecipitation

The cell lysate was successively incubated with anti-FLAG M2-Sepharose affinity gel (Sigma) for 18 h, and specific antibody for 1 h, protein A/G agarose gel (Santa Cruz Biotechnology) for 18 h. The pellets were slowly centrifuged, washed four times in lysis buffer or RIPA buffer, treated with ubiquitination or SDS-PAGE, and incubated with specific antibodies [20].

The constructed HA-ubiquitin plasmid and the indicated plasmid were transfected into gliomas cells. Cells were reacted with 25 μM proteasome inhibitor MG132 (CalBiochem, La Jolla, California) for 5 h. Cells at 48 h post transfection were lysed in RIPA buffer, immunoprecipitated with anti-ATMIN protein antibodies. The bound protein was eluted with Laemmli sample buffer and analyzed by western blot using anti-HA antibody. With cell membrane removal, proteins were re-detected with anti-ATMIN [20].

### Dual-luciferase reporter gene assay

U87 and U251 cells were co-transfected with UBR5-wild type (Wt) or UBR5-mutant type (Mut) and miR-361-5p mimic or mimic NC. After 24 h, cells were analyzed in a dual-luciferase reporter analysis system (Promega, Madison, WI, USA). Luciferase activity = firefly luciferase intensity/Renilla luciferase intensity [25].

### Tumor xenografts in nude mice

U87 cells (5 × 10^6^ cells in 100 μL PBS) transfected with si-NC and si-UBR5 were subcutaneously injected into the left axilla of male BALB/c nude mice (4 weeks old, Cell bank of Chinese Academy of Sciences, Shanghai, China). Each group was set with 8 mice. The tumor volume was measured every 3 d (volume = 1/2 × length × width^2^) [26]. The tumor tissues were paraffin-embedded, sliced to 4-μm sections, and stained with anti-Ki-67 (DAKO) for immunohistochemical staining and TUNEL staining. The image was captured with AxioVision Rel.4.6 (Carl Zeiss). The cell proliferation index was quantified by counting the proportion of Ki-67-positive cells. The apoptosis index was calculated as the percentage of TUNEL-positive cells [27].

### Statistical analysis

SPSS21.0 software (IBM, NY, USA) was employed to data analysis. All data conformed to normal distribution and homogeneous variance. Measurement data were expressed as mean ± standard deviation. Two sets of data were compared by independent sample *t* test, and data among multiple groups were compared by one-way analysis of variance (ANOVA), and Tukey’s post hoc test. Survival rate of patients was calculated by Kaplan–Meier method, univariate analysis was performed by Log-rank test, and correlation analysis was implemented with Pearson test. *P* < 0.05 was recorded as statistical difference.

## Results

### miR-361-5p and ATMIN are downregulated and UBR5 is upregulated in gliomas

**Fig. 1:**
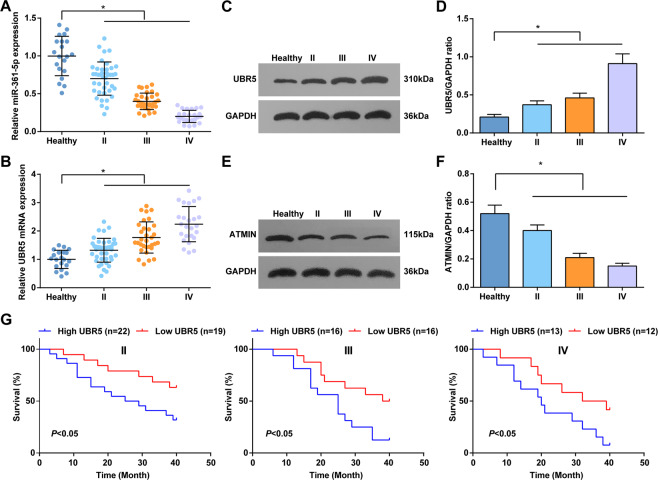
miR-361-5p and ATMIN are upregulated and UBR5 is downregulated in gliomas. **A** RT-qPCR tested miR-361-5p expression in brain tissues of healthy controls and patients with gliomas at different stages. **B** RT-qPCR tested UBR5 mRNA expression of UBR5 in brain tissues of healthy controls and patients with gliomas at different stages. **C**, **D** Western blot tested UBR5 protein expression in brain tissues of healthy controls and patients with gliomas at different stages. **E**, **F** Western blot tested ATMIN protein expression in brain tissues of healthy controls and patients with gliomas at different stages. **G** Kaplan–Meier survival curve analyzed the relationship between UBR5 expression and the prognosis of patients with gliomas at different stages, univariate analysis was performed by Log-rank test; the data were all measurement data, in the form of mean ± standard deviation; *P < 0.05 indicated the relationship between patients with gliomas at different stages.

miR-361-5p could target Twist1 to inhibit EMT of gliomas cells and presents a downregulated level in gliomas [8]. Detected by RT-qPCR, lowly expressed miR-361-5p showed in gliomas tissues (Fig. 1A). In addition, miR-361-5p level was lower in stage III tissues and the lowest in stage IV tissues, further indicating that miR-361-5p level was decreased as the tumor progressed.

Western blot detected ATMIN protein expression in patients, and the results suggested that ATMIN protein expression in glioma tissues was lower than that in normal tissues (Fig. 1E, F).

RT-qPCR and western blot measured UBR5 level and found that UBR5 was upregulated in gliomas tissues, especially in patients with stage IV gliomas (Fig. 1B–D). Thus, UBR5 level was considered to positively correlate with the WHO grade and gliomas progression. Taking the median of UBR5 mRNA expression as the cut-off value, patients with gliomas were divided into UBR5 low expression group and UBR5 high expression group and analyzed by Kaplan–Meier. The results revealed that survival rate of gliomas patients in different tumor grades with low UBR5 expression was higher (Fig. 1G) [28].

### Reduced miR-361-5p and ATIMN and enhanced UBR5 levels show in gliomas cells

**Fig. 2:**
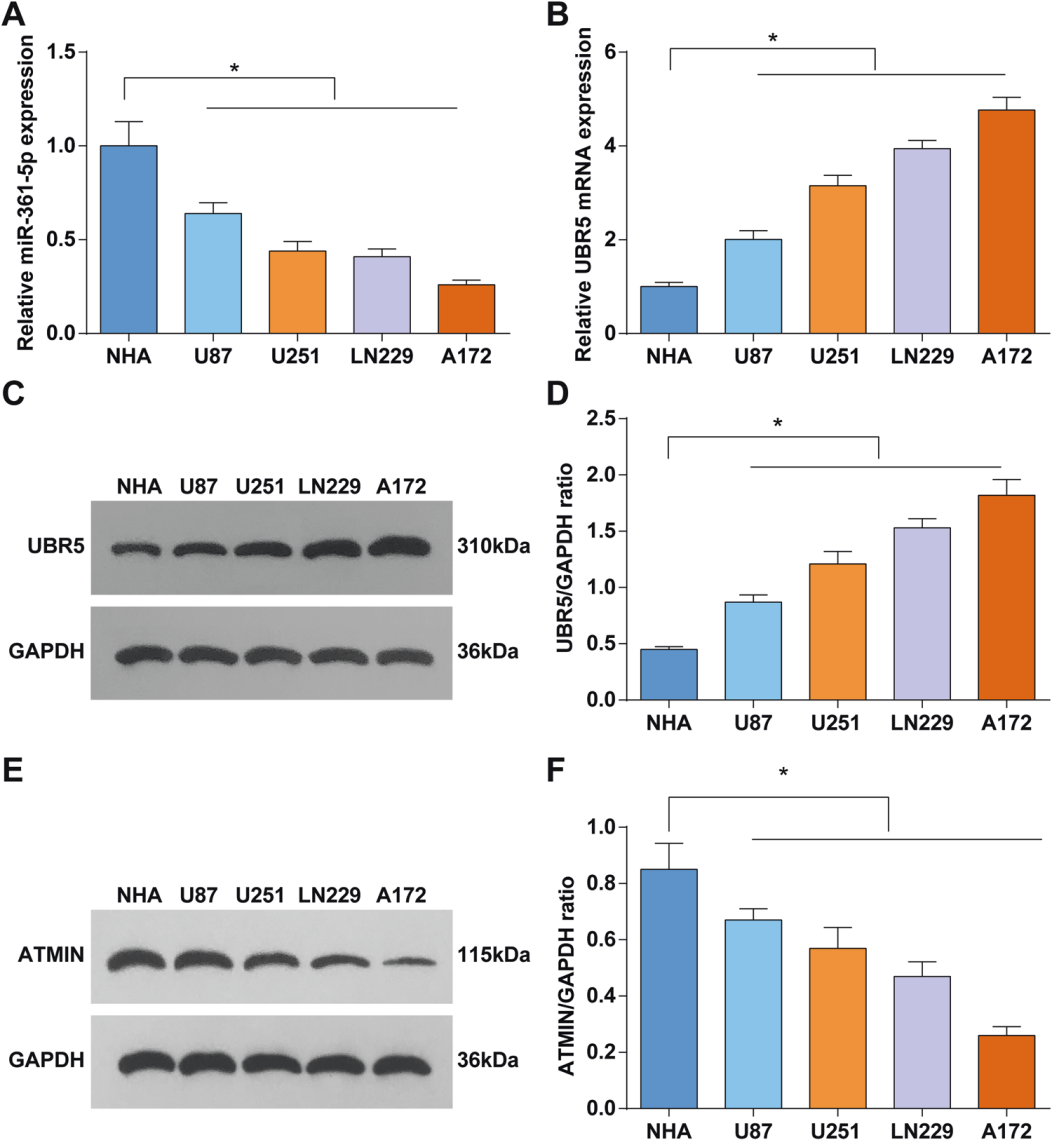
Reduced miR-361-5p and ATMIN and enhanced UBR5 levels show in gliomas cells. **A** RT-qPCR tested miR-361-5p expression in gliomas cell lines and NHA. **B** RT-qPCR tested UBR5 mRNA expression in gliomas cell lines and NHA. **C**, **D** Western blot tested UBR5 protein expression in gliomas cell lines and NHA. **E**, **F** Western blot tested ATMIN protein expression in cells. The data were all measurement data, in the form of mean ± standard deviation. *P < 0.05 compared with NHA.

To further verify the expression tendency of miR-361-5p, ATIMN, and UBR5 in gliomas, their levels in gliomas cell lines (U87, U251, LN229, and A172) and NHA were measured. As tested, miR-361-5p and ATIMN expression levels were reduced in gliomas cell lines while UBR5 level showed the opposite trend (Fig. 2A–F). Thus, U87 and U251 were screened out for follow-up experiments.

### Elevating miR-361-5p is anti-tumor for gliomas cells; downregulation of UBR5 reverses the worsening effect caused by miR-361-5p inhibition in gliomas

The biological effects of miR-361-5p on gliomas cells were explored through gain-of-function and loss-of-function experiments. At the same time, we designed rescue experiment with miR-361-5p inhibitor and UBR5 low expression.

**Fig. 3:**
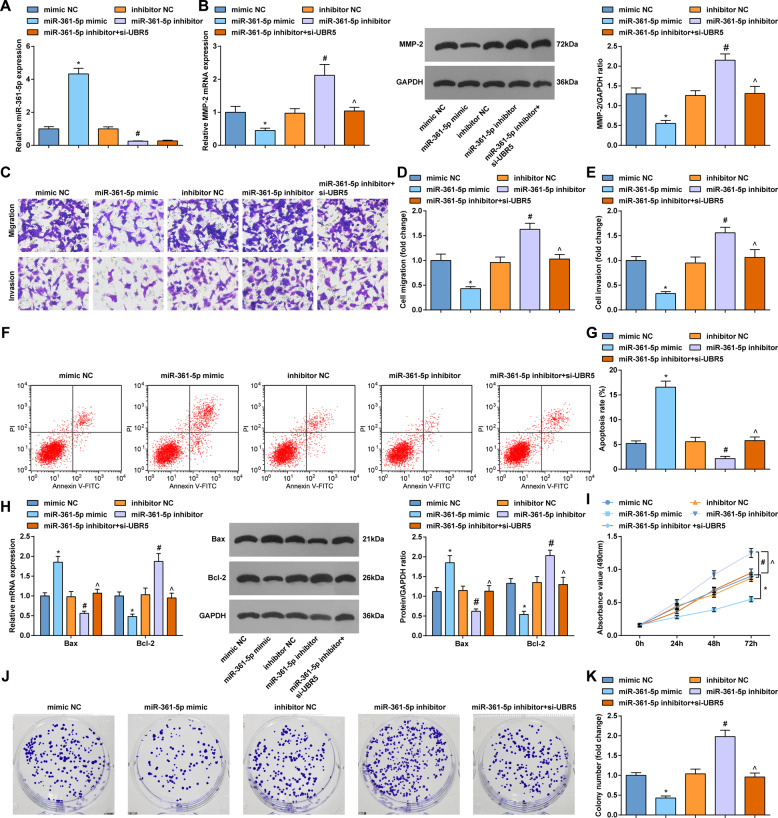
Elevating miR-361-5p is anti-tumor for gliomas cells; downregulation of UBR5 reverses the worsening effect caused by miR-361-5p inhibition in gliomas. **A** RT-qPCR tested miR-361-5p expression in U87 cells. **B** RT-qPCR and western blot tested MMP-2 expression in U87 cells. **C**–**E** Transwell tested the invasion and migration of U87 cells. **F**, **G** Flow cytometry tested apoptosis of U87 cells. **H** RT-qPCR and western blot tested Bcl-2 and Bax expression in U87 cells. **I** MTT tested the viability of U87 cells. **J**, **K** Colony formation assay tested the colony-forming ability of U87 cells; the data were all measurement data, in the form of mean ± standard deviation; *P < 0.05 compared with the mimic NC group; ^#^*P* < 0.05 compared with the inhibitor NC group; ^*P* < 0.05 compared with the miR-361-5p inhibitor group.

RT-qPCR detected the changes of miR-361-5p expression to ensure success of transfection (Fig. 3A, Supplementary Figure 1A). RT-qPCR and Western bot suggested that MMP-2 level in gliomas cells was suppressed by miR-361-5p mimic while enhanced by miR-361-5p inhibitor. Downregulation of UBR5 reversed the elevation of MMP-2 level mediated by miR-361-5p inhibitor (Fig. 3B; Supplementary Figure 1B).

Then, the effects of miR-361-5p on gliomas cell invasion, migration, apoptosis, viability, and colony-forming ability were testified by Transwell assay, RT-qPCR, western blot, flow cytometry, MTT assay, and colony formation assay. As a result of miR-361-5p overexpression, cell migration, invasion, and viability were impaired, colony number was reduced, apoptosis rate was enhanced, and Bcl-2 level was decreased and Bax level was elevated. miR-361-5p downregulation functioned in an opposite way to gliomas cell progression. Silencing UBR5 after interference reversed the effects of miR-361-5p downregulation (Fig. 3C–K; Supplementary Figure 1C-K).

### UBR5 is directly targeted by miR-361-5p

**Fig. 4:**
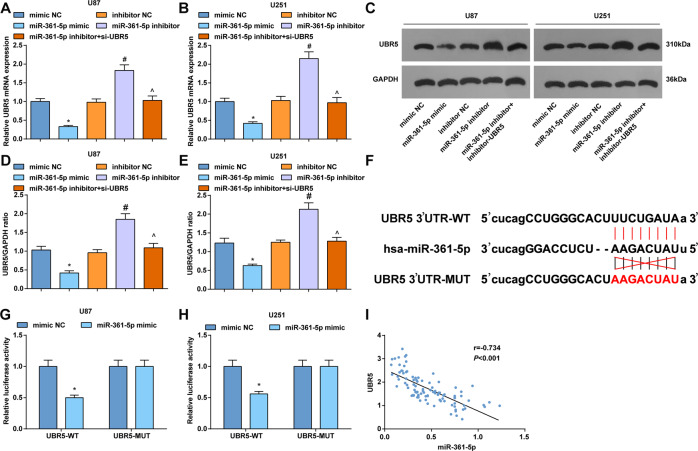
UBR5 is directly targeted by miR-361-5p. **A** RT-qPCR tested UBR5 mRNA expression in U87 cells. **B** RT-qPCR tested UBR5 mRNA expression in U251 cells. **C** Western blot tested UBR5 protein bands in U87 and U251 cells. **D** Western blot tested UBR5 protein expression in U87. **E** Western blot tested UBR5 protein expression in U251 cells. **F** Starbase predicted the binding sites of miR-361-5p and UBR5. **G** Dual-luciferase reporter gene assay verified the targeting relation of miR-361-5p and UBR5 in U87 cells. **H** Dual-luciferase reporter gene assay verified the targeting relation of miR-361-5p and UBR5 in U251 cells. **I** Correlation analysis between miR-361-5p expression with UBR5 expression in gliomas cells. The data were all measurement data, in the form of mean ± standard deviation; *P < 0.05 compared with the mimic NC group; *P < 0.05 compared with the inhibitor group; ^*P* < 0.05 compared with the miR-361-5p inhibitor group.

In gliomas cells, UBR5 levels were found to raise after miR-361-5p inhibition. On the contrary, miR-361-5p upregulation suppressed UBR5 levels. Downregulation of UBR5 suppressed the level of UBR5 mediated by miR-361-5p inhibition (Fig. 4A–E). The binding sites were found between UBR5 and miR-361-5p through Starbase website (Fig. 4F), thus miR-361-5p was speculated to target UBR5 in tumorigenesis. For further validation, results from dual-luciferase reporter gene assay demonstrated that co-transfection of miR-361-5p mimic and UBR5-Wt suppressed the luciferase activity of cells (Fig. 4G, H). Finally, we also conducted a correlation analysis and found that miR-361-5p expression was negatively correlated with UBR5 expression in clinical samples (Fig. 4I).

### UBR5 regulates ubiquitination of ATMIN

**Fig. 5:**
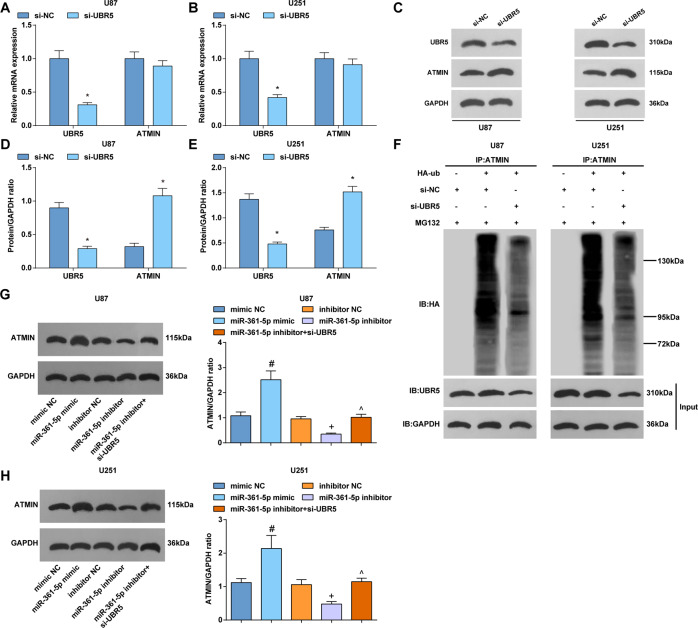
UBR5 regulates ubiquitination of ATMIN. **A** RT-qPCR tested UBR5 and ATMIN mRNA expression in U87 cells. **B** RT-qPCR tested UBR5 and ATMIN mRNA expression in U251 cells. **C** Western blot tested UBR5 and ATMIN protein bands in U87 and U251 cells. **D** Western blot tested UBR5 and ATMIN protein expression in U87 cells. **E** Western blot tested UBR5 and ATMIN protein expression in U251 cells. **F** Cells were treated with HA-labeled ubiquitin and transfected with siRNA, cells were treated with proteasome inhibitor MG132 (MG) for 2 h before lysis in denaturing buffer. Immunoprecipitation determined the ubiquitination of ATMIN. **G**, **H** Western blot detected ATMIN protein expression in U87 and U251 cells. The data were all measurement data, in the form of mean ± standard deviation; *P < 0.05 compared with the si-NC group; ^#^*P* < 0.05 compared with the mimic NC group; ^+^*P* < 0.05 compared with the inhibitor NC group; ^*P* < 0.05 compared with the miR-361-5p inhibitor group.

UBR5 ubiquitinates ATMIN during ionizing radiation to release and promotes ATM activation [13]. Based on that, the effect of UBR5 on gliomas was inferred to relate to ATMIN activity. In the present study, ATMIN protein expression manifested a reduction whereas ATMIN mRNA expression showed no change in gliomas cells after UBR5 downregulation (Fig. 5A–E), implying that UBR5 may regulate ATMIN protein through post-translational modification.

The effect of UBR5 on ubiquitination of ATMIN was determined. The endogenous ATMIN degeneration rate was measured after inhibition of protein synthesis by cycloheximide. In cells, immunoprecipitation observed the ubiquitination between UBR5 and ATMIN and finally demonstrated that knocking out UBR5 would inhibit ATMIN ubiquitination, thud elevating ATMIN expression (Fig. 5F). Moreover, the ubiquitination of ATMIN was related to its degradation, as evident by proteasome inhibitor MG132 maintaining ATMIN protein level.

Western blot detected changes in ATMIN protein expression and finally revealed that overexpression of miR-361-5p promoted while downregulation of miR-361-5p inhibited ATMIN protein expression. Knockdown of UBR5 reversed the reduction of ATMIN protein expression caused by miR-361-5p downregulation (Fig. 5G, H).

### Suppression of UBR5 retards gliomas cell growth

**Fig. 6:**
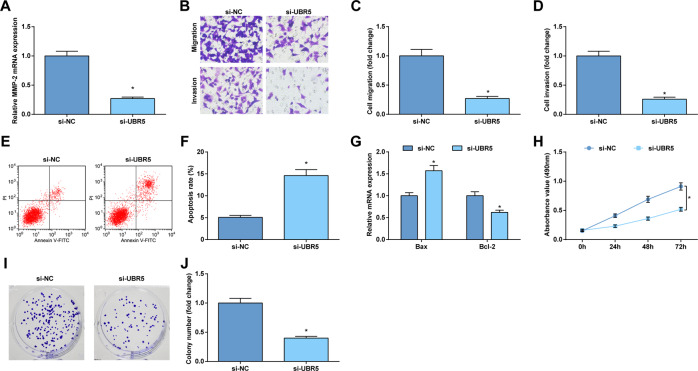
Suppression of UBR5 retards U87 cell growth. **A** RT-qPCR tested MMP-2 mRNA expression in U87 cells. **B**–**D** Transwell assay tested the migration and invasion of U87 cells. **E**, **F** Flow cytometry tested apoptosis of U87 cells. **G** RT-qPCR tested Bcl-2 and Bax mRNA expression in U87 cells. **H** MTT tested the optical density of U87 cells at 490 nm (0, 24, 48, and 72 h). **I**, **J** Colony formation assay tested the colony-forming ability of U87 cells. The data were all measurement data, in the form of mean ± standard deviation; *P < 0.05 compared with the si-NC group.

To verify the role of ATMIN ubiquitination and degradation in gliomas, UBR5 depletion was performed in gliomas cells. As demonstrated, depleting UBR5 reduced MMP-2 level in cells (Fig. 6A; Supplementary Figure 2A).

After that, the progression of U87 and U251 cells was detected in response to UBR5 depletion-induced ATMIN ubiquitination and degradation. Evidently, UBR5 depletion-induced ATMIN ubiquitination and degradation reduced the acquisition of malignant phenotype of gliomas cells (Fig. 6B–J; Supplementary Figure 2B-J).

### Downregulating UBR5 impedes gliomas tumor growth in mice

**Fig. 7:**
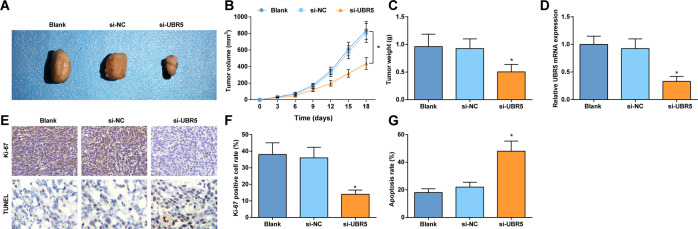
Downregulating UBR5 impedes gliomas tumor growth in mice. **A** Tumors of nude mice. **B** Tumor volume of mice. **C** Tumor weight of mice. **D** RT-qPCR detected UBR5 expression in mouse tumors. **E** Ki-67 immunohistochemical staining results (400×) and TUNEL staining (400×) of tumor tissues of mice. **F** Ki-67-positive cells in tumor tissues of mice. **G** Apoptosis rate of cell in tumor tissues of mice; *n* = 8/group; the data were all measurement data, in the form of mean ± standard deviation; *P < 0.05 compared with the si-NC group.

An in vivo model was established in mice to inspect the regulatory mechanism of UBR5 in gliomas tumor growth. U87 cells delivering downregulated UBR5 were injected into mice and greatly suppressed tumor volume and weight from the 9th day (Fig. 7A–C). RT-qPCR detected the expression of UBR5 in tumor tissues and found that UBR5 expression was reduced in mice treated with si-UBR5 (Fig. 7D). Immunohistochemical staining and TUNEL staining of tumors pictured that UBR5 knockdown decreased Ki-67 positive cells and increased cell apoptosis rate (Fig. 7E–G).

## Discussion

Malignant gliomas are most aggressive among adult central nervous system malignancies [29]. In gliomas, much attention has been paid to the relevant mechanisms in cell fate. Our experiments were designed to delve out the role of miR-361-5p in gliomas through regulating UBR5-mediated ubiquitination of ATMIN. miR-361-5p was the suppressed gene in gliomas tissues that was tightly connected with tumor grade. However, ectopic expression of miR-361-5p could limit the acquisition of malignant phenotype of gliomas cells. UBR5 was the target of miR-361-5p that was overexpressed in gliomas, and its silence was anti-tumor for gliomas tumor growth in mice. UBR5, in turn, was the mediator for ubiquitination of ATMIN which delayed gliomas development. To simplify, miR-361-5p hampered gliomas by regulating UBR5 to promote the protein expression of ATMIN (Supplementary Figure 3).

In fact, the truth that the low the miR-361-5p expression, the high the tumor grade has been discovered in gliomas, and increasing miR-361-5p level can limit cellular migration and invasion, as well as abate MMP-2 expression [7]. miR-361-5p presents an inhibited level in gliomas that partially results in the blocked apoptosis and stimulated proliferation [6]. Experimentally measured, miR-361-5p level trends to fall in gliomas and if elevated, miR-361-5p could prevent gliomas cells to migrate and invade [8]. From a novel perspective, miR-361-5p is surveyed to be sponged by COX10 antisense RNA 1, thus driving actin gamma 1-mediated proliferation and straining apoptosis of GBM cells [9]. Commonly proved, miR-361-5p is indeed the tumor suppressor in human cancers, including but not limited to gliomas. In gastric cancer and non-small-cell lung cancer, an inverse link shows between lowly expressed miR-361-5p and advanced tumor stage, and restoring miR-361-5p is devastating to cancer cell aggressive behaviors [30, 31]. In a word, miR-361-5p suppresses gliomas development, just like its anti-tumor effects in other tumors.

The targeting relation between UBR5 and miR-361-5p discovered in the present work has not confirmed by previous reports, thereby future investigations are required for verification. UBR5 is enriched in DNA damage repair-related pathway in GBM [32]. In other cancer types, such as gastric cancer, UBR5 level is also enhanced that is associated with advanced TNM stage and depleting UBR5 is the resource for cell proliferation, invasion, and migration inhibition [33]. UBR5 in gallbladder cancer maintains a higher level and repressing UBR5 in cancer cells would remarkably impair cell proliferative and colony-forming capacities [34]. Moreover, the upregulated UBR5 is connected with dismal survival of laryngeal carcinoma patients and the driving player for cell proliferation, invasion, and migration [35]. In addition to that, the pro-tumor role of UBR5 has been convinced in colorectal cancer, judged by its abundant expression in cancer tissues and cells that induces cell proliferation and tumor growth [36]. Widely discovered, UBR5 involves in cancer progression through mediating gene ubiquitination. For instance, UBR5 could promote degradation of sex-determining region Y-box 2, the tumor promoter in esophageal cancer, thereby inhibiting cell proliferation and stemness [11]. In a similar way, UBR5 was examined to promote ubiquitination of ATMIN in gliomas that is consistent with a former study [13]. If UBR5-mediated ubiquitination is suppressed, ATMIN level is tested to elevate [37], further confirming our study results. ATMIN, along with ATM, has been implied as a contributor for GBM formation [15]. Limited studies have revealed the effects of ubiquitination of ATMIN in cancer process, but there are studies mentioning the role of ATMIN in cancers. For example, ATMIN is defined as a tumor inhibitor in lung adenocarcinoma [38].

To summary, the research presents the results clear that miR-361-5p modulates the interaction of UBR5 and ATMIN to enhance ATMIN protein expression, thus to repress gliomas development in vitro and in vivo. This research from a novel angle has advanced the potential targets for gliomas treatment. However, the role of miR-361-5p/UBR5/ATMIN axis in gliomas still needs much more explorations and advancements.

## Supplementary information

supplementary Figure Legends

Supplementary figure 1.

Supplementary figure 2

Supplementary figure 3
