# Supplementary material for: MicroRNA-361-5p slows down gliomas development through regulating UBR5 to elevate ATMIN protein expression
Source: Cell Death Dis. 2021 Jul 28;12(8):746. doi: 10.1038/s41419-021-04010-1 (PMC8319180; doi:10.1038/s41419-021-04010-1)
Supplement: Supplementary file 1 — supplementary Figure Legends [file 41419_2021_4010_MOESM1_ESM.doc]

**Supplementary figure 1** Elevating miR-361-5p is anti-tumor for gliomas cells. A. RT-qPCR tested miR-361-5p mRNA expression in U251 cells; B. RT-qPCR and Western blot tested MMP-2 expression in U251 cells; C-E. Transwell tested the migration and invasion of U251 cells; F/G. Flow cytometry tested apoptosis of U251 cells; H. RT-qPCR and Western blot tested Bcl-2 and Bax expression in U251 cells; I. MTT tested the viability of U251 cells; J/K. Colony formation assay tested the colony-forming ability of U251 cells; the data were all measurement data, in the form of mean ± standard deviation; * *P* < 0.05 compared with the mimic NC group; # *P* < 0.05 compared with the inhibitor NC group; ^ *P* < 0.05 compared with the miR-361-5p inhibitor group.

**Supplementary Figure 2** Suppression of UBR5 retards U251 cell growth. A. RT-qPCR tested MMP-2 mRNA expression in U251 cells; B-D. Transwell assay tested the migration and invasion of U251 cells; E/F. Flow cytometry tested apoptosis of U251 cells; G. RT-qPCR tested Bcl-2 and Bax mRNA expression in U251 cells; H. MTT tested the optical density of U251 cells at 490 nm (0, 24, 48 and 72 h); I/J. Colony formation assay tested the colony-forming ability of U251 cells; the data were all measurement data, in the form of mean ± standard deviation; * *P* < 0.05 compared with the si-NC group.

**Supplementary Figure 3** MiR-361-5p slows down gliomas development through regulating UBR5 to elevate ATMIN expression.
