# Supplementary figures and images for: MicroRNA-361-5p slows down gliomas development through regulating UBR5 to elevate ATMIN protein expression
Source: Cell Death Dis. 2021 Jul 28;12(8):746. doi: 10.1038/s41419-021-04010-1 (PMC8319180; doi:10.1038/s41419-021-04010-1)

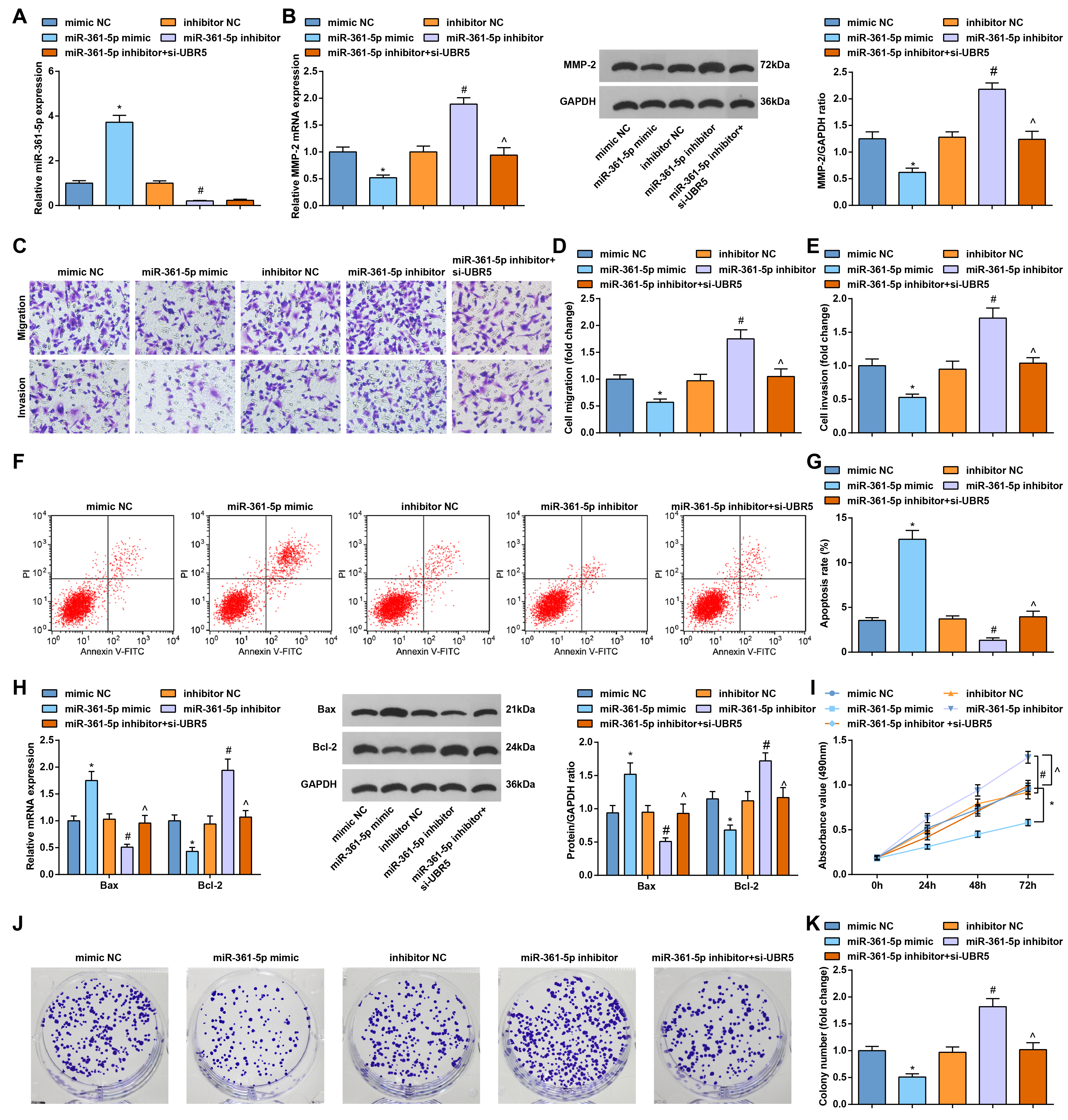

Supplement: Supplementary file 2 — Supplementary figure 1. [file 41419_2021_4010_MOESM2_ESM.tif]

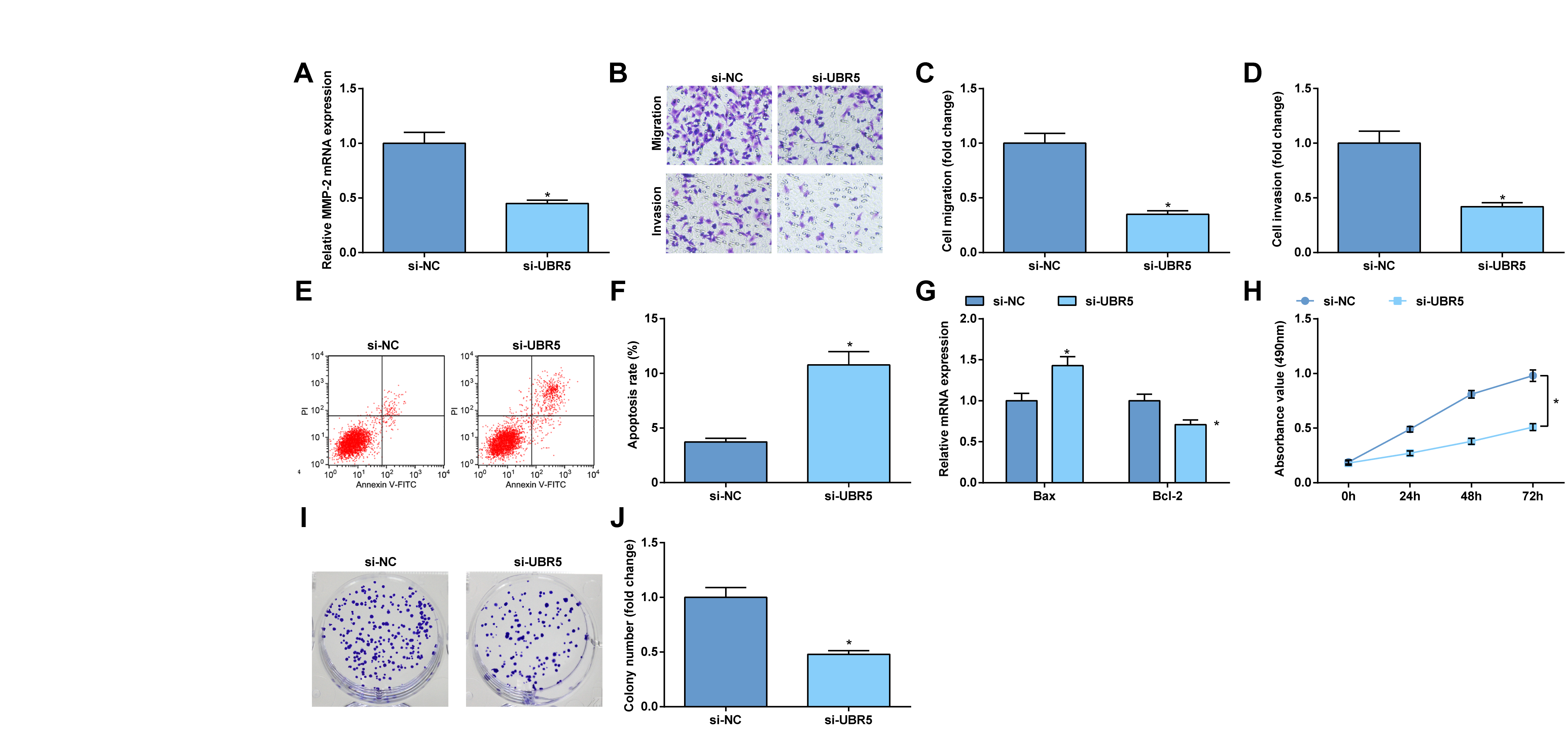

Supplement: Supplementary file 3 — Supplementary figure 2 [file 41419_2021_4010_MOESM3_ESM.tif]

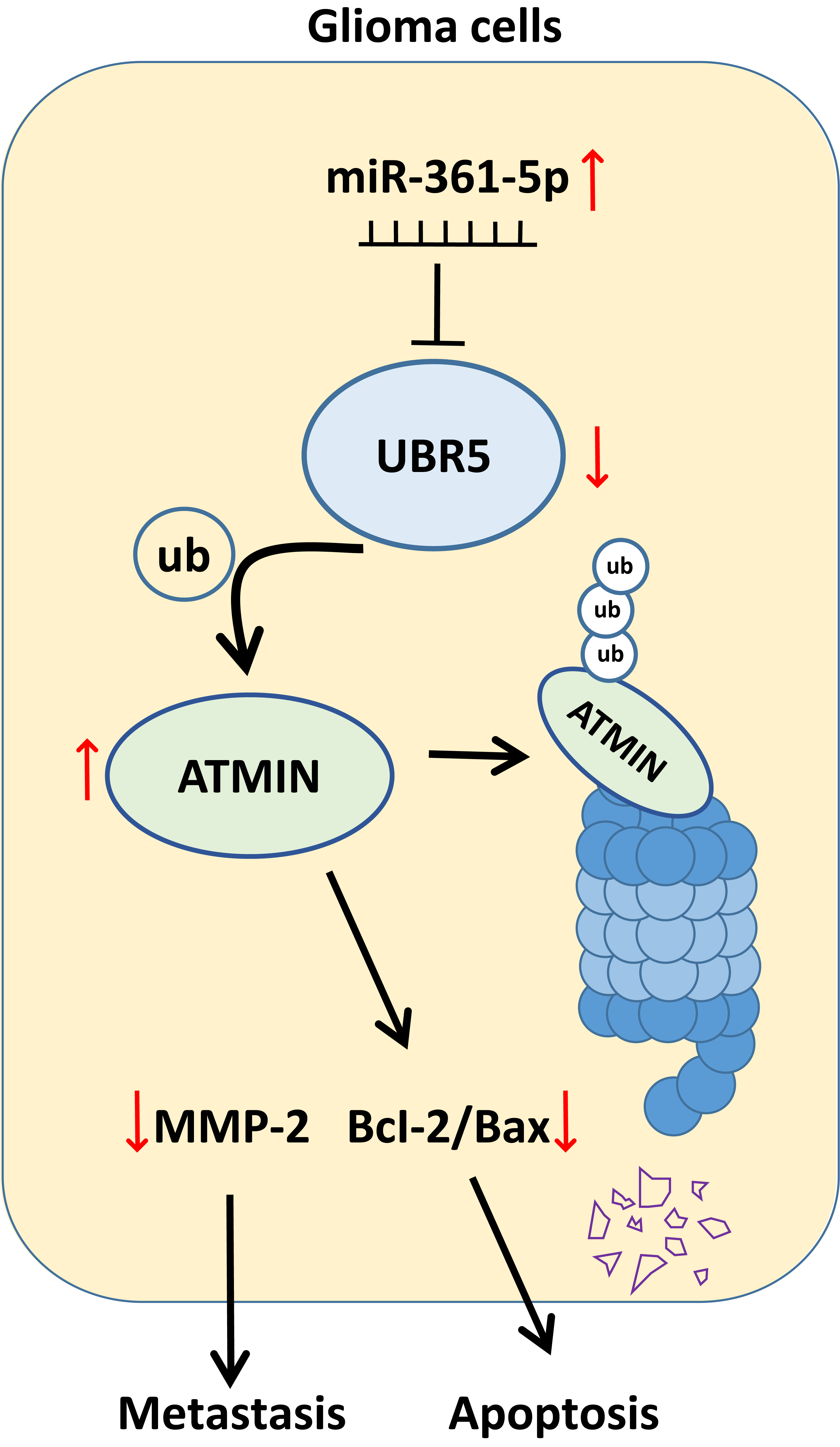

Supplement: Supplementary file 4 — Supplementary figure 3 [file 41419_2021_4010_MOESM4_ESM.tif]
